# The Memory and Affective Flexibility Task: a new behavioral tool to assess neurocognitive processes implicated in emotion-related impulsivity and internalizing symptoms

**DOI:** 10.3389/fpsyt.2025.1456691

**Published:** 2025-01-30

**Authors:** Kenneth J. D. Allen, Matthew V. Elliott, Eivind H. Ronold, Nandini A. Rajgopal, Åsa Hammar, Sheri L. Johnson

**Affiliations:** ^1^ Department of Psychology, University of California, Berkeley, Berkeley, CA, United States; ^2^ Department of Medical and Biological Psychology, University of Bergen, Bergen, Norway; ^3^ Division of Psychiatry, Haukeland University Hospital, University of Bergen, Bergen, Norway; ^4^ Department of Clinical Sciences Lund, Psychiatry, Faculty of Medicine Lund University, Lund, Sweden; ^5^ Office for Psychiatry and Habilitation, Psychiatry Research Skåne, Lund, Sweden

**Keywords:** affective flexibility, anxiety, cognitive control, depression, emotion regulation, emotion-related impulsivity, internalizing, switching

## Abstract

**Background:**

Cognitive rigidity and working memory impairment are established features of internalizing syndromes. Growing evidence suggests that deficits in *affective control* –cognitive control in the context of emotion – may underpin elevated emotion-related impulsivity in various psychiatric disorders.

**Objective:**

This study examines two components of affective control (affective flexibility and emotional working memory) as potential neurocognitive processes linking emotion-related impulsivity to internalizing psychopathology.

**Method:**

Undergraduate participants (analysis *n* = 120) completed the Memory and Affective Flexibility Task (MAFT), a novel behavioral assessment designed to assess hot cognition in affective flexibility and emotional working memory performance, alongside self-report measures of impulsivity and symptoms of internalizing disorders.

**Results:**

Structural equation modeling suggested that less accurate working memory during neutral trials (cool cognition) was associated with more symptoms of internalizing psychopathology. However, effects of hot working memory and affective flexibility were not significantly related to emotion-related impulsivity or psychopathology scores.

**Conclusions:**

Although findings provide no support for the validity of MAFT indices of hot cognition, these results replicate and extend work on the importance of cool working memory and emotion-related impulsivity as correlates of psychopathology.

## 1 Introduction

There is a growing shift in psychopathology research toward transdiagnostic approaches that transcend traditional psychiatric boundaries (1–3). These approaches aim to identify common mechanisms that underlie a wide range of psychological symptoms, offering a more integrative view of psychopathology that incorporates perspectives from neuroscience and behavioral genetics (4). Evidence continues to accumulate for the reliability and validity of transdiagnostic models of nosology (5) and treatment (6–11), underscoring their potential to reshape understanding of mental illness. Despite these promising advances, a considerable proportion of patients remain unresponsive or have considerable residual symptoms following treatment [e.g., (12)], highlighting the urgent need to identify more precise targets for effective interventions (11, 13–16).

Emotion-related impulsivity (ERI), characterized by impulsive behavior and cognition in response to heightened emotions, has emerged as a transdiagnostic factor across psychopathologies (17–23). ERI encompasses two subconstructs: (1) Feelings Trigger Action (FTA), defined as tendencies toward rash speech and action during strong emotional states; and (2) Pervasive Influence of Feelings (PIF), involving susceptibility to dysregulated cognition and motivation in response to aversive emotions (20, 24–26). Derived from factor analysis, FTA primarily includes items from the Negative and Positive Urgency scales (23, 27), whereas items comprising PIF mainly pertain to cognitive and motivational consequences of negative affect (24–26). FTA and PIF tend to be moderately correlated, consistent with theory that both involve poor constraint in the context of heightened emotion. FTA and PIF have both shown strong validity in relation to clinical outcomes (20, 28–32). In studies using path modeling and multivariate regression to consider conjoint effects, FTA better predicts externalizing syndromes and suicide attempts, whereas PIF better predicts internalizing syndromes and suicidal ideation (26, 28–32). Together, these two facets of ERI confer generalized risk for internalizing and externalizing psychopathology (20). An important next step in this line of inquiry is to better understand *how* this loss of constraint may occur.

From a neuropsychological perspective, cognitive control deficits have been theorized to contribute to ERI (17, 24), given the critical role of cognitive control in self-regulation of emotion, cognition, and goal-directed behavior (33). Latent variable models indicate that cognitive control tasks reliably map onto three core domains: inhibition, cognitive flexibility (i.e., set-shifting or task-switching), and working memory updating (34). These three domains are strongly interrelated, such that they load onto a higher-order common factor, supporting the “unity and diversity” model of cognitive control (33, 34).

Investigations into ERI and cognitive control have largely focused on response inhibition (35). Such studies suggest that the effects of FTA (and its constituent Urgency scales) on standard metrics of motor response inhibition are weak outside of clinical populations (36). Less work has examined how ERI relates to cognitive control processes other than response inhibition. This gap is surprising given that deficits in working memory and shifting are found across psychiatric diagnoses (22, 37, 38). In the only study of ERI and cognitive flexibility that we have identified, the authors relied on a self-report measure of the latter construct that corresponds poorly with behavioral indices of switching (39). Some evidence suggests an association of ERI with poorer working memory performance (40, 41), yet null findings have also emerged (42).

In studies that have considered hot cognition, several studies have shown that deficits in one form of affective control– emotional response inhibition –are strongly associated with FTA and Urgency [ (43–48, but also see (49)]. Comparatively little is known, however, about other facets of affective control, such as emotional flexibility and emotional working memory, in relation to ERI. This gap deserves empirical attention for several reasons. It is well-established that working memory and flexibility are adversely influenced by heightened stress (50, 51). Indeed, meta-analytic work suggests that working memory may be the facet of cognitive control most vulnerable to stress (50, 51). Furthermore, working memory performance is disrupted by emotional information processing in clinical populations – with most evidence derived from samples with internalizing psychopathology (52–54). Although fewer studies are available, lower affective flexibility has been reported among those with psychological symptoms (55), including those with anxiety (56, 57), depression (58–60), as well as processes shown to increase risk for internalizing disorders, such as worry (57) and rumination (61). Hot working memory and affective flexibility are a natural focus for exploration of processes underlying ERI and internalizing psychopathology.

Much of the available neuropsychological literature has focused on FTA and the Urgency scales. The behavioral correlates of PIF, in contrast, have received less empirical attention. Despite this discrepancy, PIF has shown stronger relationships with internalizing symptoms (31), rumination (30), and suicidal ideation severity (28, 62). While it is plausible that FTA and PIF are both linked to disruption in affective flexibility *and* emotional working memory (17, 39, 40), dysfunction in emotional working memory may have special relevance for PIF. This is because PIF has been shown to be tied to more problematic responses to even low levels of stress (30). Given the sensitivity of working memory to stress (50), we hypothesized that PIF, as compared to FTA, would uniquely relate to poorer hot working memory. This prediction of a stronger tie of hot working memory with PIF than FTA is also consistent with the stronger ties of PIF with internalizing disorders, which are tied to hot working memory.

**Figure 1:**
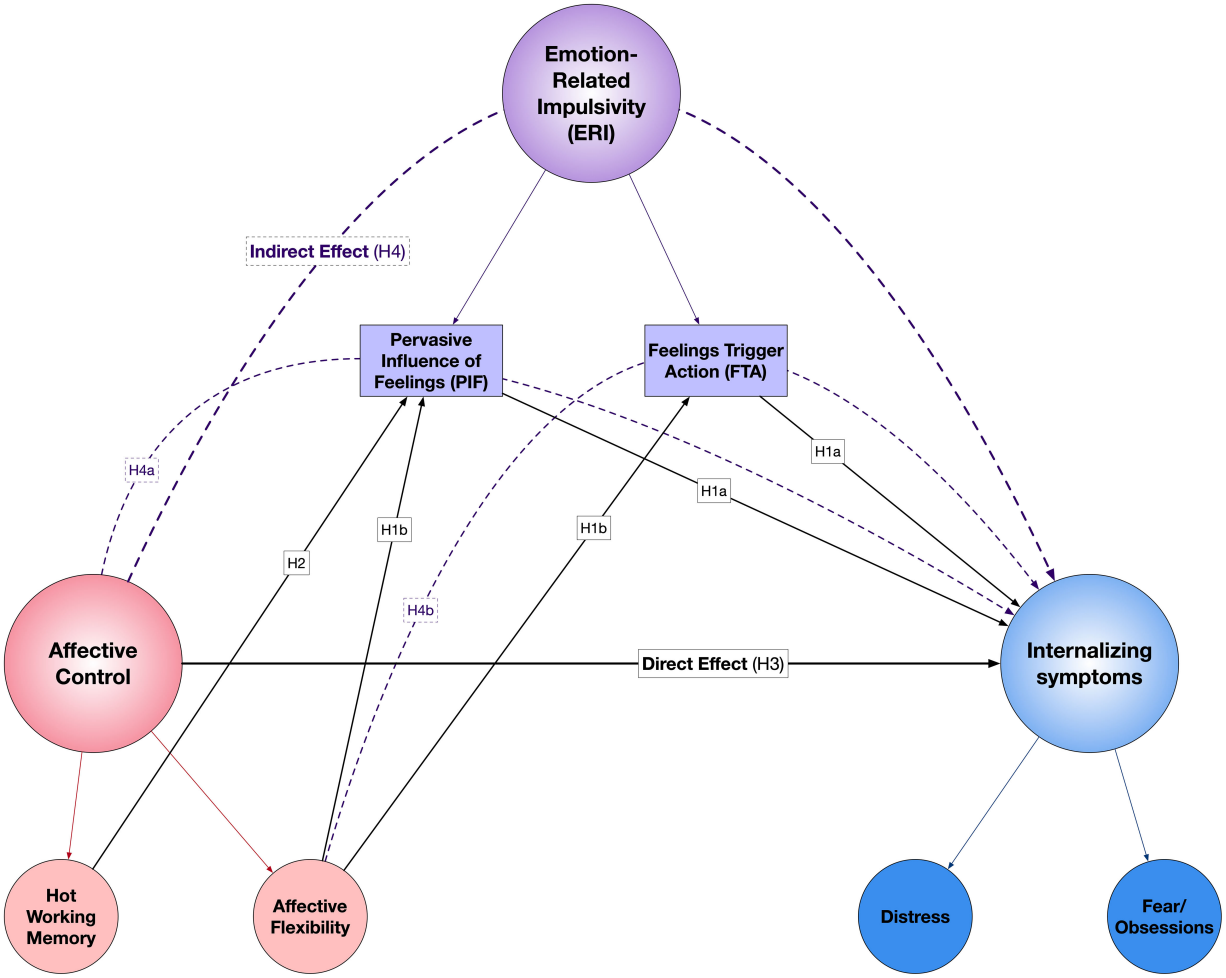
Neurocognitive Model of Emotion-Related Impulsivity and Internalizing Psychopathology.

The current study aims to address two major gaps in the literature bridging ERI, cognitive/affective control, and internalizing conditions. First, we provide novel evidence considering hot and cool cognition, and of working memory and affective flexibility, conjointly. Second, we consider PIF and FTA conjointly. We theorize that affective control processes partially drive relationships between ERI and internalizing psychopathology (see **Figure 1**).

In service of these aims, we sought to develop and validate a novel behavioral assessment tool, the Memory and Affective Flexibility Task (MAFT) to index affective flexibility and emotional working memory. To our knowledge, this is the first task designed to concurrently evaluate these two key affective control processes. We designed the task, though, by integrating features of well-established paradigms commonly used to assess working memory and cognitive flexibility: the *n*-back (63) and task-switching (64).

Based on our proposed model of ERI and its neurocognitive underpinnings in relation to internalizing symptoms (**Figure 1**), the present study tests the following hypotheses (see attached preregistration materials), seeking to establish convergent and divergent validity of the MAFT as a novel behavioral assay of affective flexibility and emotional working memory, as well as to extend prior work implicating affective control as a key factor explaining the robust links between ERI and psychopathology:

Hypothesis 1. Both facets of ERI (PIF and FTA) will be independently associated with (a) higher levels of internalizing symptoms and (b) diminished affective flexibility on the MAFT.

Hypothesis 2. PIF will also be associated with worse emotional working memory performance on the MAFT.

Hypothesis 3. MAFT performance on indices of affective flexibility and emotional working memory will be inversely associated with internalizing symptoms [independent of ERI; see (65)].

Hypothesis 4. Multivariate structural equation modeling (SEM) will reveal parallel indirect statistical effects of (a) affective flexibility and emotional working memory on internalizing symptoms through PIF and (b) affective flexibility on internalizing symptoms through FTA. Stated differently, we expect that emotional working memory will show indirect effects on internalizing only through PIF.

In addition to (a) replicating prior studies indicating a particularly strong link between PIF and symptoms of internalizing disorders, and (b) extending previous work implicating emotional response inhibition in FTA and psychopathology, this research will further elucidate the role of distinct affect-related inhibitory processes with plausibly greater relevance to the *cognitive* component of ERI (i.e., PIF) and its relationship with internalizing symptoms associated with depression and anxiety.

## 2 Materials and methods

All study procedures were approved by the University IRB before data collection began. An earlier version of the hypotheses and analyses is available in the attached preregistration materials.

### 2.1 Participants and procedures

The study sample comprised of 130 undergraduate students aged 18-47 years from a major public university who received course credit for their involvement (see **Table 1**). Ten participants were excluded for multiple indicators of poor attention (e.g., failure on at least 2 out of 3 “attention check items”, performance below chance on the MAFT), leaving an analysis sample of 120. As shown in **Table 1**, *n*’s vary slightly by measure. Scores for questionnaires were coded as missing for one participant who failed to correctly respond to at least two out of three “attention check” items embedded in self-report questionnaires. MAFT switch scores were coded as missing for 4 participants who attained < 50% accuracy across the positive and negative switch trials. All participants in the sample attained < 50% omission error rates on MAFT *n-*back trials. One participant did not complete the IDAS-II.

**Table 1:** Demographic characteristics (N = 120).

	*M* or *n*	*SD* or %
Age	21.45	4.2
Education	14.45	1.36
Gender		
Female	103	86.67%
Male	15	12.50%
Nonbinary	1	0.83%
Race/Ethnicity		
American Indian or Alaskan Native	2	1.67%
Asian	54	45.00%
Black or African	1	0.83%
Hispanic, Latino/a, or Spanish	13	10.83%
Middle Eastern or North African	3	2.50%
White	29	24.17%
Mixed race or Multiracial	16	13.33%
Not reported/Other	2	1.67%

*M*, Mean; *SD*, Standard Deviation.

Participants predominantly identified as female and endorsed a fairly diverse racial/ethnic background that was generally representative of the broader student population. We recruited interested individuals from a departmental research participant pool based on responses to an online prescreening survey, which included the Urgency scale of the abbreviated UPPS-P (66) to oversample individuals with elevated ERI. Students who reported elevated Urgency (i.e., scores greater than 3.5 out of 5) were actively invited to participate. To capture the full range of ERI, recruitment was also fully open to other students in the research participation program. Following an online assessment via Qualtrics software (67), participants who passed embedded attention checks were invited to a 2.5-hour laboratory session, in which they provided written informed consent before completing a series of neuropsychological assessments, including the MAFT, and several other tasks not considered here.

### 2.2 Measures

#### 2.2.1 Three-Factor Impulsivity Index

The Three-Factor Impulsivity Index (TFII; 25) is a 54-item self-report measure that evaluates three latent factors of impulsivity derived from factor analysis. These latent factors comprise the aforementioned subordinate facets of ERI, *Feelings Trigger Action* (FTA) and *Pervasive Influence of Feelings* (PIF), as well as *Lack of Follow Through* (LFT), a third construct representing trait impulsivity independent of emotion. TFII respondents rate statements about reflexive behavioral (FTA) and cognitive (PIF) reactivity to emotions, as well as about impulse control unrelated to affect (LFT), on a Likert scale from 1 (“I disagree A LOT”) to 5 (“I agree A LOT”). Research supports the TFII’s robust three-factor structure, which demonstrates high internal consistency (McDonald’s *ω* = 0.90-0.95 in this sample) as well as shared and unique associations with psychiatric disorders and physical exercise (68), underscoring its validity as a comprehensive measure of impulsivity (25). Consistent with prior work, TFII scores in this sample were moderately intercorrelated, with the strongest association observed between the two facets of ERI.

#### 2.2.2 Revised Inventory of Depression and Anxiety Symptoms

The revised Inventory of Depression and Anxiety Symptoms (IDAS-II; 71) is a 99-item questionnaire designed to assess the frequency and severity of symptoms across the internalizing spectrum. Respondents are asked to indicate the extent to which they have experienced each symptom over the preceding two weeks, on a five-point scale from “Not at all” to “Extremely”. The Factor analyses of the IDAS-II have consistently identified latent dimensions of *Distress*, *Fear/Obsessions, and Well-Being* (69–72). Here, we focus on Distress and Fear/Obsessions, as the core symptom domains of the internalizing disorders.

#### 2.2.3 Memory and Affective Flexibility Task (MAFT)

**Figure 2:**
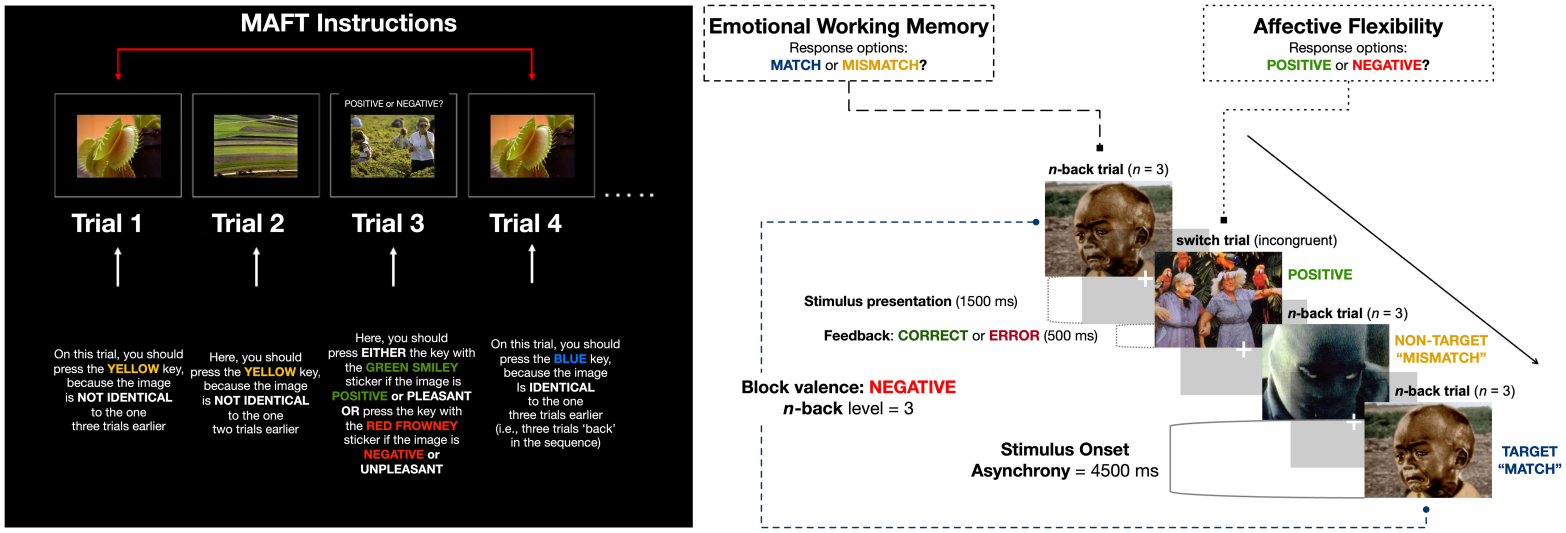
Memory and Affective Flexibility Task (MAFT) Schematic.

The MAFT (**Figure 2**) is a novel, timed, computer-based, behavioral assessment designed to measure emotional working memory and affective flexibility. On each MAFT trial, participants were instructed to respond via keypress to an image from the International Affective Picture System [IAPS; (73)]. Participants had to determine their response based on the two main trial types, which probed working memory and affective flexibility. The working memory (i.e., “n-back”) trials followed standard n-back procedures, during which participants were asked to respond via keypress whether a given image was identical (a “match”) or not (“mismatch”) to a target image shown n trials earlier (where n = 1-3) in the sequence. On affective flexibility (i.e., “switch”) trials, participants were instead asked to respond via keypress according to the emotional valence of the presented image as either “positive” or “negative.”

Text instructional cues distinguished he working memory vs. switch trial types. On “n-back” trials, IAPS images were shown without any text. On “switch” trials, the text “POSITIVE or NEGATIVE?” appeared above the IAPS image. All IAPS images were presented for 1500 milliseconds (ms), and participants were given a 4500 ms window to respond (equivalent to the trial duration/stimulus onset asynchrony; see **Figure 2**). Participants used four keys in total to respond – “Match,” “Mismatch,” “Positive,” and “Negative.”The MAFT consisted of 198 trials separated into nine experimental blocks. Participants completed three, valence-specific, blocks – positive, negative, neutral – for each level of n-back difficulty (n = 1, 2, and 3-back trials). Each block contained 20 + n trials to account for the number of “mismatch” stimuli shown before the first possible target “match” stimulus. Before proceeding to the experimental blocks, participants were asked to complete two initial practice blocks with trial-level feedback (presented for 500 ms). The practice blocks were repeated until the participant achieved an accuracy threshold of at least 70% on n-back trials at the n=1 and n=2 levels (11 trials at the n = 1 condition and 12 trials in the n = 2 condition).

Within each block, ~60% of trials were n-back trials (i.e., 12 + n trials); four were “match” trials and the rest were “mismatch” trials. The emotional valences of all n-back trials were congruent with the block type. The remaining 8 trials, ~40%, were switch trials of alternating emotional valence. N-back and switch trials were interspersed in pseudo-random order to minimize predictability.

For each trial, an image was drawn randomly from the IAPS stimulus battery without replacement. The Negative (reverse-coded for valence comparison) and Positive image sets were approximately matched on standardized ratings of arousal (negative M = 5.94, SD = 0.77; positive M = 5.22, SD = 1.02) and valence intensity (negative M = 7.22, SD = 1.04; positive M = 7.15, SD = 0.79); Neutral images were chosen for their comparatively low arousal (M = 2.88; SD = 0.57) and intermediate valence ratings (M = 4.98; SD = 0.30). The MAFT was programmed by the lead author for implementation in Inquisit 6.0 stimulus presentation software (74) and is available upon request.

The MAFT yielded accuracy and reaction time performance metrics that could be parsed by emotional valence and n-back difficulty. Accuracy for n-back trials was calculated as the proportion of correct “match” trial hits and “mismatch” trial rejections. Accuracy for switch trials was calculated as the proportion of positive IAPS stimuli classified as positive and negative IAPS stimuli classified as negative. Reaction time (RT) was calculated as the average speed of correct responses, for both n-back and switch trials. Before calculating RT, we excluded trials in which a person responded too quickly to be considered a genuine reaction to the stimulus (< 200 ms) and those without a response within the 4500 ms window. Then, Z-scores were generated for each participant and trial-level outlier RTs (> |3|) were trimmed.

Although our major focus was on emotional working memory and affective flexibility across positive and negative emotional valence, switch trials with neutral images were included with the goal of providing additional information about evaluation of stimuli without salient emotional content. We hoped to consider the percentage of neutral IAPS stimuli (on switch trials) classified as negative as an index of subjective interpretive bias, rather than accuracy. Overall, neutral stimuli on switch trials were primarily categorized as “positive” (*M* = 0.64, *SD =* 0.11), consistent with previous research using a parallel index from the emotional stop-signal task [see (44)]. As noted by one reviewer, though, the forced choice of assigning negative or positive ratings to these neutral pictures is problematic. We do not consider these scores further here.

After developing the task design, pilot work was conducted to evaluate the speed of trials, the number of trials to feasibly incorporate to avoid fatigue, and the clarity of instructions. The task was adjusted in small ways after each round of this informal feedback.

#### 2.2.4 Statistical analyses

We used JASP version 0.19.1 (75) for all statistical analyses and assumption checks, as well as Python version 3.12.4 (76) for data cleaning and processing before hypothesis-testing.

Next, we performed analyses for hypothesis-testing. Here, we acknowledge several deviations from our pre-registered statistical approach (see attached). Primarily, we incorporated more comprehensive pre-processing procedures at a trial level, and by considering overall accuracy rates, rather than adhering to our original plan to remove outliers defined at +/-2.5 SDs from the mean. We made this decision to preserve the ability to consider valid individual differences in task performance. Second, we reduced analytic repetition by incorporating planned regressions into a broader structural equation modeling (SEM) framework. Third, we relied on factor scores rather than narrower scales from the IDAS-II.

**Figure 3:**
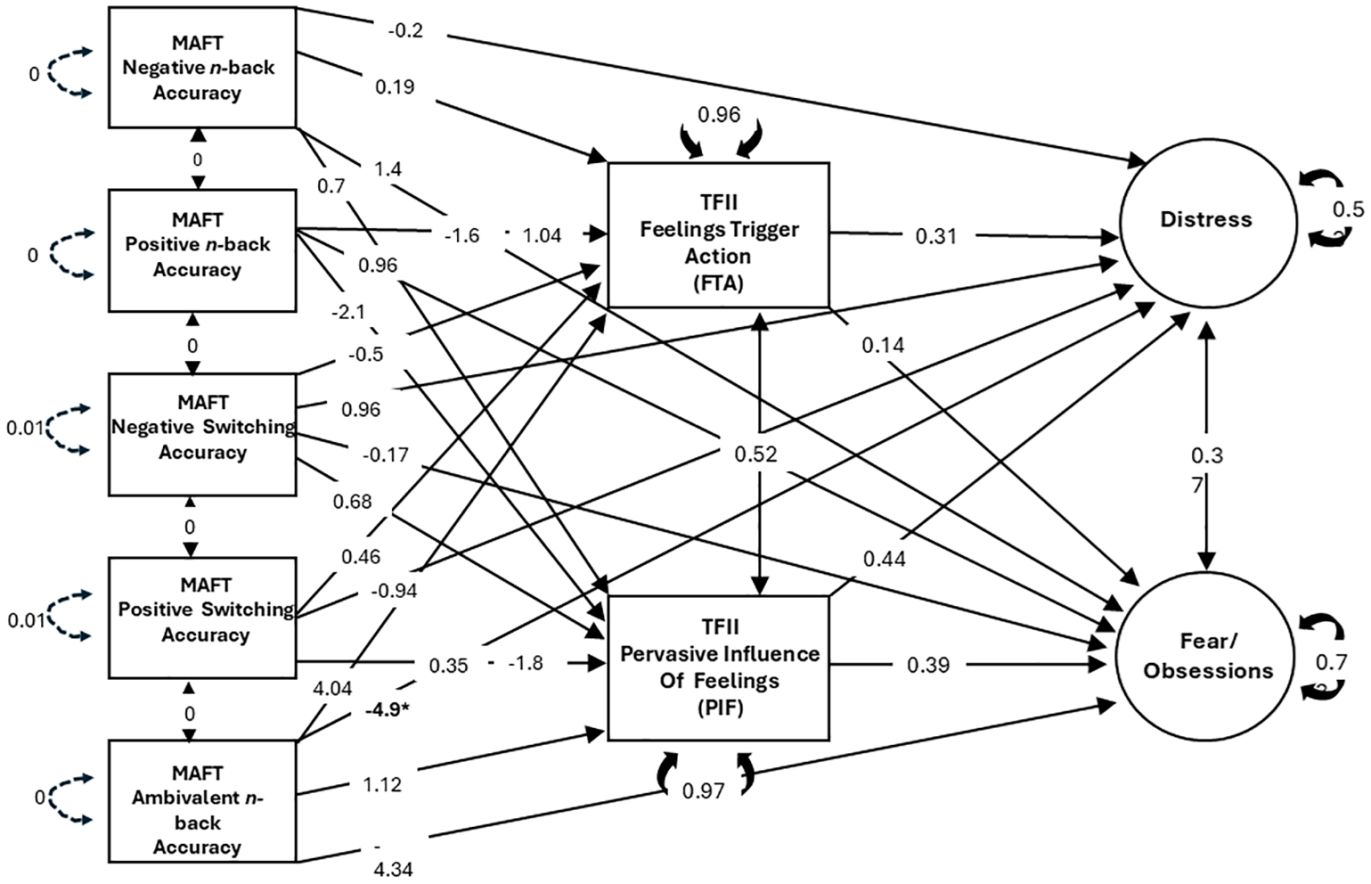
Structural equation model of affective control, ERI, and internalizing symptoms. Standardized regression coefficients shown (β). *p<.05.

We used SEM to test our proposed conceptual model of ERI, MAFT metrics of affective control, and internalizing symptoms from the IDAS-II (**Figures 1**, **3**). Bivariate correlations of key variables were performed as preliminary analyses. Our SEM model used robust calculation of standard errors, which are less influenced by outliers and heteroscedasticity. Variables were standardized before entry, and we used full information maximum likelihood (FIML) to impute missing values. To examine hypothesized pathways of affective flexibility and emotional working memory through FTA and PIF, we calculated parameter estimates associated with indirect effects of MAFT metrics on internalizing dimensions. Significant indirect effects would suggest that some portion of the relationship between two variables (e.g., affective inflexibility and depression) is explained by shared variance between the predictor, an intermediary variable (e.g., ERI), and the outcome (e.g., depression). We focus on the specific regression coefficients as tests of our mediational model hypotheses.

## 3 Results

Descriptive statistics for key study variables are provided in **Table 2**. The distributions for most of the MAFT summary metrics approximated normality, with two exceptions: Switch accuracy scores for negative stimuli were negatively skewed and leptokurtic, and negative response bias scores were also leptokurtic.

**Table 2:** Descriptive statistics for key variables (N = 120).

	*M* (*SE*)	Range	Skewness (*SE =* 0.22)	Kurtosis (*SE =* 0.44-0.45)
Memory and Affective Flexibility Task (MAFT)				
MAFT *n*-Back Accuracy (%)				
*Negative*	0.88 (0.005)	0.71 – 1.00	0.63	0.74
*Positive*	0.88 (0.005)	0.69 – 1.00	0.88	1.69
*Neutral*	0.94 (0.004)	0.82 – 1.00	0.29	0.54
MAFT Switch Accuracy (%)				
*Negative*	0.90 (0.010)	0.25 – 1.00	-2.57	11.69
*Positive*	0.92 (0.007)	0.62 – 1.00	-1.39	2.39
MAFT *n-*Back Reaction Time (ms)				
*Negative*	1084.88 (16.85)	750.62 – 1599.55	0.67	0.04
*Positive*	1051.54 (15.88)	662.87 – 1635.13	0.81	1.04
*Neutral*	1048.21 (13.79)	660.77 – 1492.06	0.42	0.42
MAFT Switch Reaction Time (ms)				
*Negative*	1201.11 (15.16)	867.50 – 1887.75	0.80	1.84
*Positive*	1176.58 (14.42)	858.36 – 1757.75	0.69	0.98
Three-Factor Impulsivity Index (TFII)				
Feelings Trigger Action (FTA)	2.91 (0.09)	1.11 – 4.88	0.09	0.27
Pervasive Influence of Feelings (PIF)	3.53 (0.09)	1.08 – 5.00	-0.40	-0.74
Revised Inventory of Depression and Anxiety Symptoms (IDAS-II)				
Distress	125.37 (3.59)	31.58 – 266.51	0.67	1.18
Fear/Obsessions	51.06 (1.68)	14.47 – 113.67	0.86	0.59

*SE*, Standard Error.

Valid MAFT switching data *n* = 116, valid TFI *n* = 119, valid IDAS-II *n* = 118.

### 3.1 MAFT performance

**Figure 4:**
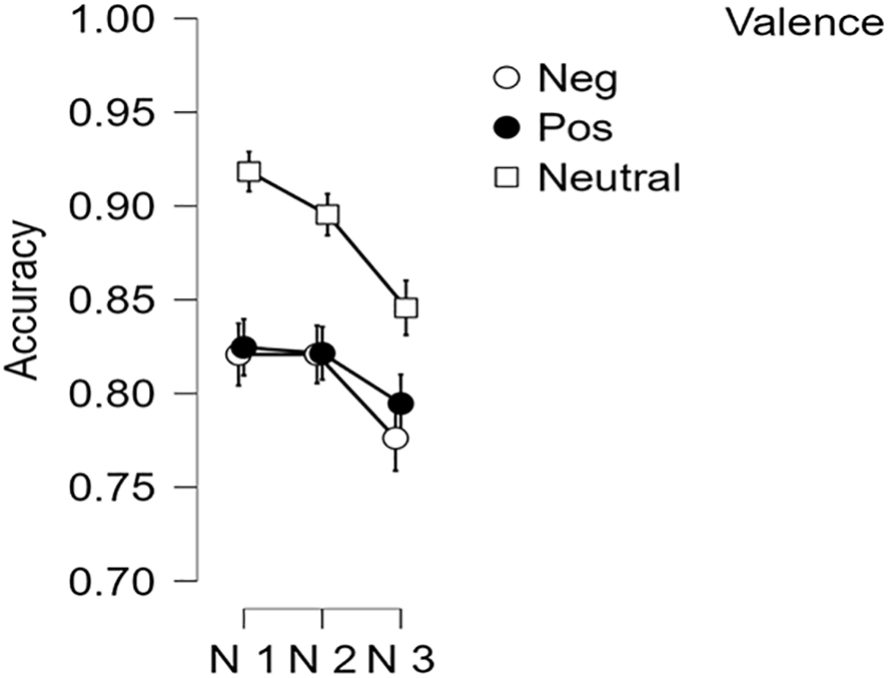
N-Back Accuracy by n-level and stimulus valence. Error bars = +/- 1 standard error.

Accuracy of working memory performance across different *n-*back levels and valence categories is depicted in **Figure 4**. As expected, participants demonstrated poorer accuracy on *n*-back trials as working memory demand (i.e., the *n*-level) increased, *F*(1.79, 173.97) = 9.634, *p* < 0.001, *η* ^2^ = 0.01.

**Figure 5:**
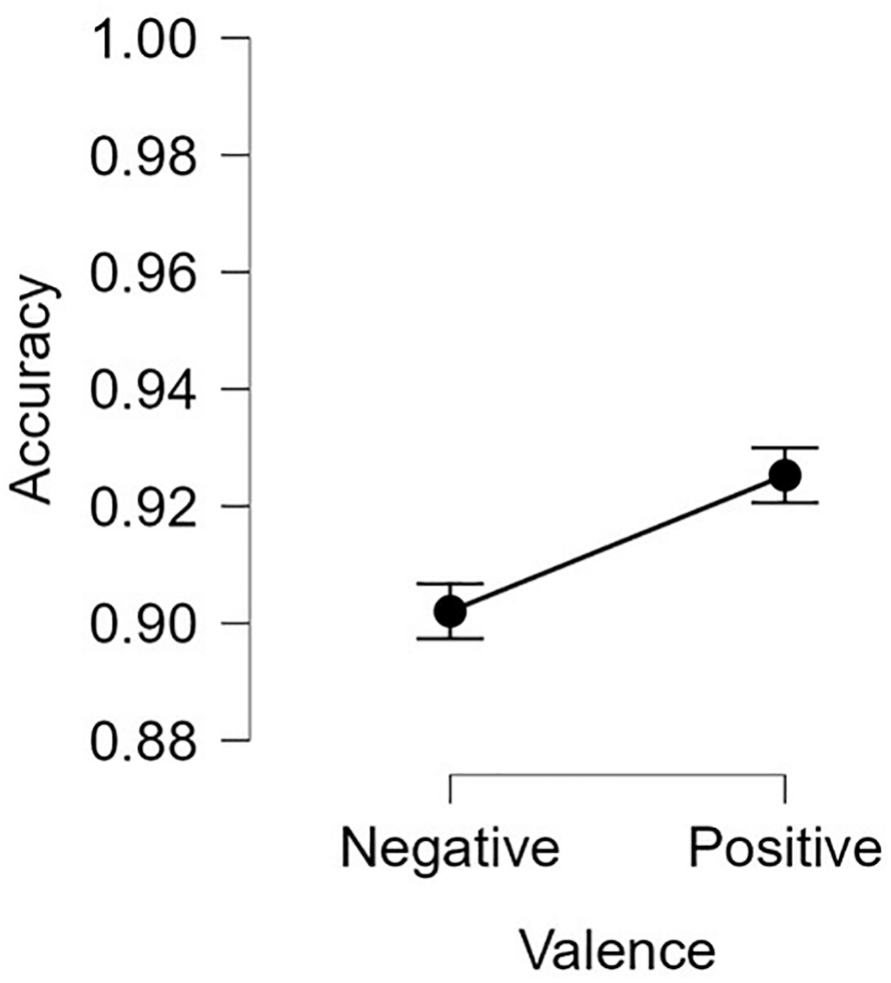
Switch accuracy by Valence. Error bars = +/- 1 standard error.

Accuracy rates were lower for emotional *n-*back trials with negative and positive images as compared to those with neutral stimuli, *F*(1.93, 219) = 45.52, *p* < 0.001, *η* ^2^ = 0.277, as **Table 2** and **Figure 4** demonstrate. Participants were also less accurate when prompted to classify negative images relative to positive stimuli on switch trials, *F*(1, 115) = 6.14, *p* < 0.05, *η* ^2^ = 0.051 (**Figure 5**).

**Figure 6:**
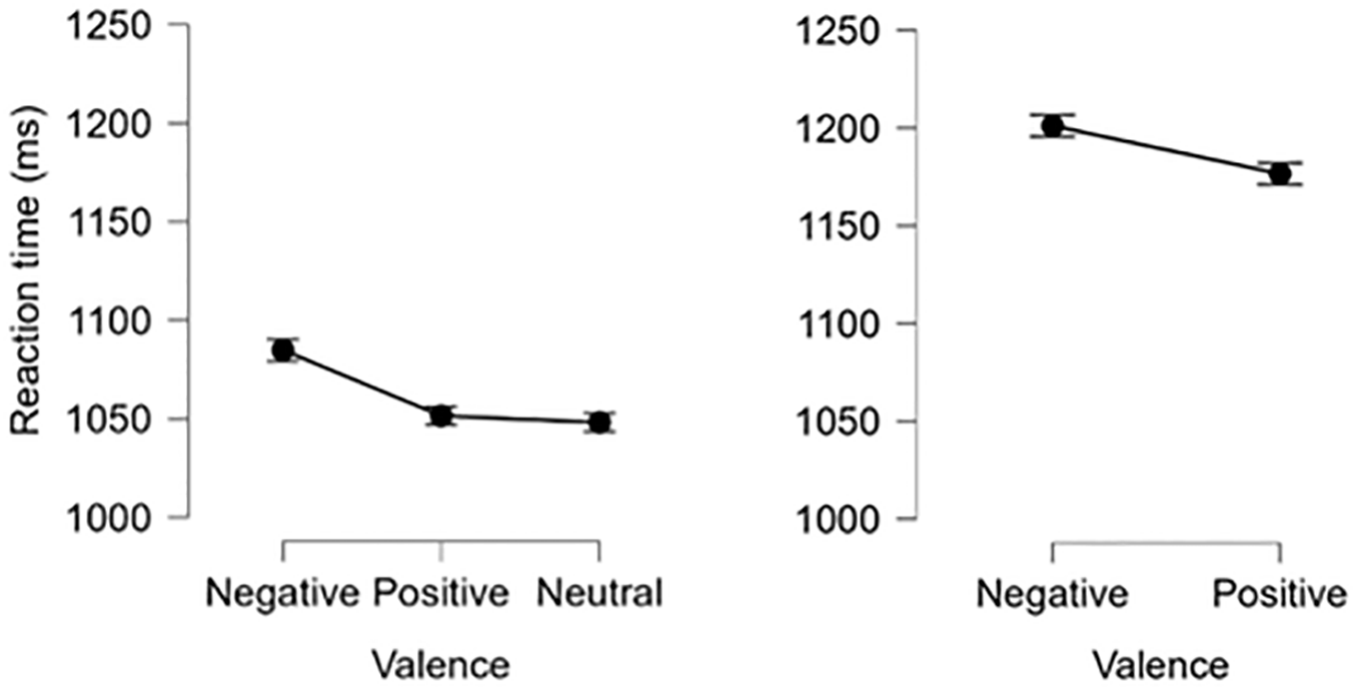
Reaction time by stimulus valence for (left) n-Back and (right) switch conditions. Error bars = +/- 1 standard error.

**Figures 6A, B** portray the relatively slower response speed for negative stimuli in n-back and switch trials. Participants had significantly slower RTs for negative *n*-back trials compared to those with positive and neutral stimuli, *F*(1.87, 119)^1^ = 11.19, *p* < 0.001, *η* ^2^ = 0.09 (**Figure 6A**). Similarly, switch trial RTs were significantly slower when evaluating negative images relative to positive ones, *F*(1, 115) = 9.89, *p* < 0.01, *η* ^2^ = 0.08 (**Figure 6B**).

Intercorrelations among MAFT summary metrics are presented in **Table 3**. Because Shapiro-Wilks tests suggested non-normality, Spearman’s correlations were used. Consistent with the hypothesized separability of these indices, most accuracy scores showed small to moderate correlations. We observed a small but significant association between accuracy for positive and neutral *n*-back trials. In addition, accuracy on the positive switch trials was correlated significantly with switch accuracy on the negative trials, and with accuracy on *n*-back trials with negative and neutral stimuli. All other intercorrelations among accuracy scores were small and nonsignificant.

**Table 3:** Bivariate Spearman correlation coefficients.

	1	2	3	4	5	6	7	8	9	10	11	12	13
*n*-Back Accuracy (*n* = 120)													
1. Negative	–												
1. Positive	0.21^*^	–											
2. Neutral	0.22^*^	0.22^*^	–										
Switch Accuracy (*n* = 116)													
3. Negative	0.16	0.12	0.01	–									
4. Positive	0.32^***^	0.17	0.16^*^	0.42^***^	–								
*n-*Back Reaction Time (*n* = 120)													
5. Negative	0.01	-0.10	-0.14	0.04	0.10	–							
6. Positive	-0.04	-0.11	-0.11	0.00	0.08	0.82^***^	–						
7. Neutral	0.01	-0.08	-0.13	0.04	0.15	0.81^***^	0.87^***^	–					
Switch Reaction Time (*n* = 116)													
8. Negative	-0.10	-0.13	-0.20*	-0.03	-0.04	0.66^***^	0.66^***^	0.69^***^	–				
9. Positive	-0.16	-0.13	-0.16	-0.07	0.03	0.71^***^	0.68^***^	0.68^***^	0.85^***^	–			
Three-Factor Impulsivity Index (TFII; *n* = 119)													
10. FTA	0.07	-0.05	0.12	0.04	0.06	-0.13	-0.07	-0.08	-0.17	-0.12	–		
11. PIF	0.09	-0.12	0.02	0.12	0.04	-0.01	0.02	-0.01	0.00	-0.02	0.52^***^	–	
Revised Inventory of Depression and Anxiety Symptoms (IDAS-II; *n* = 118)													
12. Distress	0.02	-0.09	-0.11	0.08	-0.04	-0.04	-0.01	-0.06	-0.05	-0.05	0.44^***^	0.61^***^	–
13. Fear/Obsessions	0.03	-0.05	-0.08	-0.06	-0.11	0.04	0.00	-0.05	0.00	0.04	0.30^**^	0.44^***^	0.68^***^

FTA, Feelings Trigger Action; PIF, Pervasive Influence of Feelings. ^*^ *p* < 0.05, ^**^ *p* < 0.01, ^***^ *p* < 0.001.

Before testing hypotheses, we considered whether age or gender might be confounders in MAFT performance. Bivariate Spearman correlations indicated no significant effects of age or gender, all *r*’s ¾.17, *p*’s >.05.

Somewhat surprisingly, bivariate correlations suggested no speed-accuracy tradeoff in MAFT performance. However, RT indices were highly correlated across all MAFT summary metrics of emotional working memory and affective flexibility (**Table 3**; all *r*’s > 0.65), suggesting that RT scores may be more indicative of individual differences in processing or motor response speed than specific influences of task condition. Accordingly, we calculated an overall mean RT summary score across *n*-back and switch trials. Because this overall MAFT RT index was not significantly correlated with any of the MAFT accuracy indices or the TFI scores, *r*’s ¾ |.14|, *p*’s >.05. we did not consider RT further in multivariate analyses.

### 3.2 Multivariate effects of MAFT metrics on ERI and psychopathology

We used an SEM framework to test core predictions (particularly Hypothesis 4) derived from our proposed neurocognitive model of ERI associated with internalizing psychopathology. Specifically, we constructed a structural model to examine direct effects of MAFT performance metrics on internalizing-related symptoms of Distress and Fear/Obsessions, as well as the indirect effects of these same observed behavioral indicators on psychopathology through FTA and PIF from the TFII (see **Figures 1**, **3**).

As preliminary descriptive analyses before conducting SEM, we performed bivariate correlations of MAFT indices with ERI and psychopathology, provided in **Table 3**. As shown, we did not find significant bivariate associations of MAFT performance summary metrics with ERI or psychopathology scores.

Consistent with prior work, ERI was robustly related to the IDAS-II Distress score and more modestly but significantly associated with the IDAS-II Fear/Obsessions score. The correlation with Distress was stronger for PIF than for FTA (*Z* = 2.22, *p* = 0.02), again replicating previous findings on ERI in relation to internalizing symptoms. The strength of the correlation with Fear/obsessions did not differ significantly for PIF vs. FTA (*Z* = 1.74, *p* = 0.08).

**Figure 3** depicts the results of the SEM-based mediation analysis, which we used to test our theoretical model of how ERI might statistically explain hypothesized effects of affective control on internalizing psychopathology (also see **Figure 1**). Paths for ERI to psychopathology were significant, consistent with hypothesis 1A. Nonetheless, contrary to all other hypotheses, results did not support any links of the MAFT hot working memory or affective flexibility scores with either ERI or psychopathology. As shown in **Table 4**, the neutral working memory scores were the only MAFT variable that showed a significant direct effect, to IDAS-II Distress scores.

**Table 4:** Parameter estimates from the SEM model.

*Direct effects*								
							95% Confidence Interval	
			Estimate	Std. Error	z-value	p	Lower	Upper
nBack neg	→	Distress	-0.024	1.233	-0.019	0.985	-2.441	2.393
nBack pos	→	Distress	1.004	1.220	0.823	0.411	-1.387	3.395
switch neg	→	Distress	0.962	0.516	1.866	0.062	-0.049	1.973
switch pos	→	Distress	-0.944	0.884	-1.068	0.285	-2.676	0.788
nBack neu	→	Distress	-4.912	1.787	-2.749	0.006	-8.415	-1.410
nBack neg	→	Fear/Obs	1.395	1.218	1.146	0.252	-0.991	3.782
nBack pos	→	Fear/Obs	0.407	1.508	0.270	0.787	-2.549	3.364
switch neg	→	Fear/Obs	-0.165	0.697	-0.237	0.813	-1.530	1.201
switch pos	→	Fear/Obs	-1.832	1.165	-1.572	0.116	-4.115	0.452
nBack neu	→	Fear/Obs	-4.336	2.293	-1.891	0.059	-8.830	0.159

*Indirect effects*										
									95% Confidence Interval	
					Estimate	Std. Error	z-value	p	Lower	Upper
nBack neg	→	FTA	→	Distress	0.058	0.452	0.128	0.898	-0.829	0.945
nBack neg	→	PIF	→	Distress	0.311	0.699	0.444	0.657	-1.059	1.681
nBack pos	→	FTA	→	Distress	-0.491	0.524	-0.938	0.348	-1.517	0.535
nBack pos	→	PIF	→	Distress	-0.940	0.783	-1.200	0.230	-2.474	0.595
switch neg	→	FTA	→	Distress	-0.015	0.338	-0.044	0.965	-0.678	0.648
switch neg	→	PIF	→	Distress	0.302	0.373	0.812	0.417	-0.428	1.033
switch pos	→	FTA	→	Distress	0.143	0.405	0.353	0.724	-0.650	0.936
switch pos	→	PIF	→	Distress	0.154	0.527	0.293	0.770	-0.879	1.187
nBack neu	→	FTA	→	Distress	1.247	0.646	1.929	0.054	-0.020	2.514
nBack neu	→	PIF	→	Distress	0.493	0.829	0.595	0.552	-1.132	2.118
nBack neg	→	FTA	→	Fear/Obs	0.027	0.210	0.128	0.898	-0.386	0.439
nBack neg	→	PIF	→	Fear/Obs	0.273	0.605	0.451	0.652	-0.913	1.459
nBack pos	→	FTA	→	Fear/Obs	-0.228	0.257	-0.886	0.375	-0.733	0.276
nBack pos	→	PIF	→	Fear/Obs	-0.825	0.700	-1.179	0.238	-2.197	0.547
switch neg	→	FTA	→	Fear/Obs	-0.007	0.157	-0.044	0.965	-0.315	0.301
switch neg	→	PIF	→	Fear/Obs	0.266	0.330	0.804	0.421	-0.382	0.913
switch pos	→	FTA	→	Fear/Obs	0.066	0.188	0.353	0.724	-0.302	0.435
switch pos	→	PIF	→	Fear/Obs	0.135	0.464	0.292	0.770	-0.774	1.045
nBack neu	→	FTA	→	Fear/Obs	0.580	0.494	1.172	0.241	-0.389	1.549
nBack neu	→	PIF	→	Fear/Obs	0.433	0.744	0.582	0.560	-1.025	1.891

*Total effects*								
							95% Confidence Interval	
			Estimate	Std. Error	z-value	p	Lower	Upper
nBack neg	→	Distress	0.345	1.522	0.227	0.821	-2.638	3.328
nBack pos	→	Distress	-0.427	1.625	-0.263	0.793	-3.612	2.758
switch neg	→	Distress	1.250	0.797	1.568	0.117	-0.313	2.813
switch pos	→	Distress	-0.647	1.278	-0.506	0.613	-3.152	1.858
nBack neu	→	Distress	-3.172	2.090	-1.518	0.129	-7.269	0.925
nBack neg	→	Fear/Obs	1.695	1.351	1.255	0.210	-0.953	4.343
nBack pos	→	Fear/Obs	-0.646	1.757	-0.368	0.713	-4.090	2.798
switch neg	→	Fear/Obs	0.094	0.649	0.144	0.885	-1.179	1.367
switch pos	→	Fear/Obs	-1.630	1.276	-1.277	0.202	-4.131	0.872
nBack neu	→	Fear/Obs	-3.323	2.329	-1.426	0.154	-7.889	1.243

*Total indirect effects*								
							95% Confidence Interval	
			Estimate	Std. Error	z-value	p	Lower	Upper
nBack neg	→	Distress	0.369	0.978	0.377	0.706	-1.548	2.285
nBack pos	→	Distress	-1.431	1.145	-1.249	0.212	-3.676	0.814
switch neg	→	Distress	0.288	0.640	0.449	0.653	-0.967	1.542
switch pos	→	Distress	0.297	0.836	0.355	0.722	-1.342	1.937
nBack neu	→	Distress	1.740	1.218	1.429	0.153	-0.646	4.127
nBack neg	→	Fear/Obs	0.300	0.718	0.418	0.676	-1.107	1.706
nBack pos	→	Fear/Obs	-1.053	0.838	-1.257	0.209	-2.696	0.589
switch neg	→	Fear/Obs	0.259	0.445	0.582	0.561	-0.613	1.130
switch pos	→	Fear/Obs	0.202	0.599	0.337	0.736	-0.971	1.375
nBack neu	→	Fear/Obs	1.013	0.938	1.079	0.280	-0.826	2.852

Robust standard errors, robust confidence intervals, ML estimator.

Fear/Obs, Fear/Obsessions. MAFT scores are accuracy scores. Neg, Negative; Neu, Neutral; Pos, Positive; FTA, Feelings Trigger Action; PIF, Pervasive Influence of Feelings.

→ denotes the effect on.

Given the limited statistical power, we conducted *post-hoc* Bayes correlations to examine the links of the MAFT variables with the ERI and psychopathology indices. No correlations yielded Bayes factors > 10.

## 4 Discussion

In the current study, we developed and provided preliminary validity tests for the Memory and Affective Flexibility Task (MAFT), a new behavioral assessment task designed to index key aspects of affective control. We designed the MAFT designed to provide metrics of working memory and switching performance, under both cool and hot (i.e., emotional) conditions. We employed this task to provide an integrative test of links between these two facets of cognitive control, ERI, and core dimensions of internalizing psychopathology. Our work is novel in integrating two forms of ERI, and two forms of cognitive control, both of which were assessed across trials with neutral, positive, and negative stimuli. Our reliance on an SEM model to disentangle common and unique cognitive processes embedded in the MAFT scores is a strength of our approach. We begin by considering our findings regarding the effects of ERI on psychopathology. Then, we discuss analyses specific to the MAFT and evidence in support of this new task. Finally, we turn to the integrative model findings.

As shown in bivariate correlations (**Table 3**) and in our SEM model (**Figure 3**), our results replicated and extended previous work indicating robust correlations between ERI and psychopathology. Relatively less literature has considered the Pervasive Influence of Feelings (PIF) form of ERI, which focuses on unconstrained cognitive and motivational responses to negative emotions. Consistent with prior findings (20), we found that PIF was more powerfully tied to distress-related internalizing symptoms compared to scores on the Feelings Trigger Action (FTA) scale, which focuses rash speech and behavior in response to emotions. This work was also novel in considering the effects of ERI on psychopathology using the IDAS-II, a comprehensive inventory of internalizing symptoms, which allowed us to consider distress versus fear/obsessions within the same model. As expected, ERI effects generalized across both forms of internalizing symptoms, consistent with previous work highlighting the robust transdiagnostic role of ERI in various psychiatric conditions. Nonetheless, effects of ERI on fear/obsessions were relatively modest. Recent work has suggested that ERI may be more relevant for obsessive symptoms when other risk variables are present, such as intolerance of ambiguity (77). Future work might consider the contexts in which ERI is particularly related to specific forms of psychopathology.

Turning to the MAFT, several findings preliminarily supported the validity of this novel task. Most individuals were able to perform the task at above chance levels. As expected, MAFT emotional working memory performance significantly diminished as the value of *n* increased, suggesting strong parallels with previous research using similar *n-*back designs (63, 78). Our task was constructed to allow direct comparison between the effects of positive and negative stimuli on working memory performance – a key addition to the literature. Although many previous studies have examined the effect of negative stimuli on working memory performance, we are aware of only two investigations that have conjointly considered negative and positive stimuli on working memory, and those yielded inconsistent effects; Levens and Phelps (79) found that positive and negative stimuli both were related to enhanced accuracy in a recency probes task, whereas Rączy and Orzechowski (80) found that positively valenced words, but not negatively valenced words, interfered with accuracy on an two-back task. We observed lower accuracy in working memory performance on the *n*-back in the context of both positive and negative trials as compared to neutral trials. Our detection of interference during negative trials might reflect our use of emotional pictures, as contrasted with the work by Rączy and Orzechowski (80). Our findings for non-specific interference from emotionally arousing stimuli of either valence further align with previous research on the susceptibility of working memory to acute stress (50).

Regarding affective flexibility, accuracy on the switch trials was modestly correlated with accuracy on the working memory trials, indicating that the two trial types might capture partially separable processes. Of concern, though, accuracy scores for the negative switch trials were highly leptokurtic, suggesting that as constructed, the MAFT did not effectively elicit sufficient variability in switching performance. Future versions could potentially benefit from manipulating difficulty levels, perhaps by adapting task demands to require more rapid responses. In considering the relatively high-performance levels, it is worth noting that participants may have been able to accurately respond by considering their affective state, rather than attending carefully to the stimulus and task demands; future versions of the task might include conditions in which a participant is asked to respond to specific features embedded within affective images. Computational methods to extract specific processes tied to task performance are also recommended.

Although researchers have varied in their use of accuracy versus speed as indices of flexibility, our task design allowed us to examine RT across *n-*back and switch trial conditions. With this novel information, the performance profile we observed calls into question whether RT can be interpreted as a valid index of affective flexibility. Response speed was highly correlated across *n*-back and switch trials, with all RT index correlations >.65. This profile suggests that it is more likely that RT indices on MAFT switch trials are indicative of processing and/or motoric speed than they are specific to flexibility. These findings highlight an unexpected benefit of considering working memory and flexibility within the same task. As has been indicated in some prior work across cognitive tasks [e.g., (54)], negatively valenced stimuli led to a slowing of RTs on both working memory and switching trials. This is consistent with the idea that negative information could lead to a slowed, more cautious approach that may be nonspecific to working memory or switching demands.

Taken together, our analyses of task conditions suggest partial success in achieving our aim of creating a novel affective control task. That is, we found evidence that MAFT *n*-back trial performance tracked as expected with the level of working memory load, and we observed interference effects of emotion stimuli on working memory task performance. We also saw appropriate separation between *n-*back and switch trial scores. Armed with this information, we used MAFT accuracy indices to test our hypotheses concerning ERI and psychopathology.

Contrary to prediction, bivariate correlations showed no significant effect of MAFT performance on indices of ERI or psychopathology, and our multivariate SEM model provided no evidence that the hot working memory or affective flexibility scores were related directly or indirectly to ERI or to psychopathology scores (see **Figure 3**). Although few studies are available, it is worth noting that our findings are conceptually consistent with those of a previous study, which showed that working memory in the context of a stress manipulation was unrelated to ERI (81).

In contrast to the null effects for affective control in the our SEM model, we observed a direct effect of cool working memory dysfunction (i.e., lower accuracy on *n*-back trials with neutral images) on the IDAS-II Distress score. This is consistent with a large body of previous work that those who struggle with cool cognitive control facets may be at higher risk for internalizing syndromes (22). We extend this body of research by showing that the indirect effect of cool working memory on Distress through ERI was not significant, suggesting that cool working memory and ERI show separable effects on Distress. This is consistent with recent models highlighting that working memory and ERI may have unique genetic pathways toward psychopathology (82).

In sum, the current study provides three findings that contribute to the understanding of ERI, cognition, and psychopathology. First, we replicated and extended previous work linking ERI to internalizing psychopathology. Second, our multivariate model provided support for the importance of cool working memory for internalizing symptoms. Third, our findings indicated that ERI and cool working memory may exert separable effects on psychopathology.

Nonetheless, our findings provided little support for the hot cognition parameters from the MAFT, and we acknowledge that even the effect for cool working memory was relatively modest. Although consistent with previous work attempting to link self-rated and behavioral task performance metrics of conceptually related constructs (83, 84), several limitations specific to the MAFT suggest the need for caution in interpreting results from this novel task. As described, few people obtained low negative switch accuracy scores, and RT scores were strongly tied to individual differences, and less to task-specific demands. Given all effects for hot cognitive indices in relation to psychopathology and impulsivity were null, there is some question of whether the task adequately activated emotion. The MAFT relies on a very common approach in hot cognition research of using brief presentations of valenced stimuli. Any emotion elicitation effects of such brief stimulus presentations may be minor, and aside from subtle reaction time changes, we have no evidence to verify that an emotion powerful enough to interfere with processing was evoked. The addition of psychophysiology indices could help validate the extent of emotion arousal induced by the valenced stimuli. Overall, the reliability and validity of the novel MAFT task remain largely unestablished.

Other limitations are less specifically tied to the MAFT. Our cross-sectional design constrains our ability to comment on the direction of effects. We relied on self-rated measures of symptom severity, and it will be important to consider how findings generalize to diagnostic indicators. Our sample was limited to undergraduate students, although here it is worth noting that undergraduate students now demonstrate a prevalence of diagnosable psychological disorders that is comparable to the prevalence observed in the general population (85). Most of our sample was female, and there is a need to assess generalizability of effects across genders. Given higher rates of impulsivity in clinical and male groups, these sample issues may have limited ability to detect behavioral indicators of impulsivity. Perhaps most critically, our sample size of 120 is quite small given that some suggest sample sizes of 250 for testing SEM path models. Our small sample may have hindered the ability to detect meaningful effects and may limit the replicability of effects. Regarding replicability, though, we would note that not only were the bivariate and multivariate effects for most MAFT parameters on ERI and psychopathology null, but effect sizes were small as well. Bayes correlations also did not support meaningful effects of MAFT indices with psychopathology or impulsivity. Nonetheless, findings from the study should be interpreted with caution until replicated in a larger sample.

Overall, given the relatively limited evidence to support the MAFT affective control indices, future research would do well to test how parameters on the MAFT correspond to those obtained using traditional, stand-alone working memory and switching tasks. Such work would ideally include testing a large sample and considering the effects of clinical disorders.

Despite the limitations, current findings are consistent with the large body of work on cognitive remediation for psychopathology and provide particular support for interventions focused on working memory. Here, we raise one caveat though. The relatively small effect sizes of the cognitive tasks on the clinical outcomes suggest the need to consider comprehensive interventions that pair cognitive remediation with other clinical approaches (86, 87). We hope that ongoing work will integrate basic research into transdiagnostic clinical intervention approaches.

## Data Availability

The raw data supporting the conclusions of this article will be made available by the authors, without undue reservation.
